# Microscopic Polyangiitis in Systemic Sclerosis

**DOI:** 10.1155/2010/148528

**Published:** 2010-08-09

**Authors:** Hiroshi Hashimoto

**Affiliations:** ^1^Juntendo University School of Medicine, 2-1-1 Hongo, Bunkyoku, Tokyo 113-0033, Japan; ^2^Division of Rheumatology, Aiwakai Medical Corporation, 1-22-23 Kamiyouga, Setagayaku, Tokyo 158-0098, Japan

## Abstract

AAV in SSc is described from the point of view of MPA. Some of reported SSc cases with AAV are thought to exhibit the characteristic clinical manifestations of MPA, although ANCA positivity in SSc is uncommon. MPA is clinically characterized by a multisystemic disease such as RPGN, pulmonary hemorrhage, mononeuritis, and skin involvement, as well as other manifestations in conjunction with high levels of inflammatory activity such as high ESR or CRP. It is also characterized by a high frequency of MPO-ANCA, showing predominant pANCA by IIF. When rapid renal failure or RPGN with active urine sediments, pulmonary hemorrhage and/or systemic inflammatory manifestations are observed in patients with SSc having positive ANCA, the possibility of MPA should always be considered. If SSc patients with MPA have life-threatening visceral involvement such as the above clinical manifestations, the patients should be treated with induction therapy using cyclophosphamide, methotrexate, corticosteroids, or plasmapheresis, etc. according to the severity of the disease soon after the diagnosis of MPA. It is important not to overlook characteristic clinical manifestations of AAV during the course of the disease in SSc in order to diagnose MPA early.

## 1. Introduction

Systemic sclerosis (SSc), which mostly affects middle-aged women, is a chronic disorder of connective tissue characterized by inflammation and fibrosis and by degenerative changes in the blood vessels, skin, synovium, skeletal muscle, and certain internal organs, notably the gastrointestinal tract, lung, heart, and kidney [1]. The life-threatening visceral involvements in SSc include scleroderma renal crisis, pulmonary hypertension, and lung fibrosis.

 On the other hand, microscopic polyangiitis (MPA), which mostly affects elderly people, is a systemic disease characterized by vasculitis involving small blood vessels, particularly the glomerular and pulmonary capillaries, and serologically by antineutrophil cytoplasmic antibody (ANCA) positivity [2–4]. MPA is also well known to present as one form of primary ANCA-associated vasculitis (AAV) as well as Wegener's granulomatosis (WG) [2]. Clinical manifestations in this disease include rapidly progressive glomerulonephritis (RPGN) and pulmonary hemorrhage, which are life-threatening visceral involvements. 

 Cases of SSc patients with AAV have been reported, although ANCA positivity in SSc is uncommon. Some of these cases are thought to exhibit the characteristic clinical manifestations of MPA. In this paper, AAV in SSc will be described from the point of view of MPA.

## 2. ANCA and MPA

 ANCA is well known to be associated with small-sized vasculitic disorders such as MPA, WG, and allergic granulomatous angiitis (AGA) in primary systemic vasculitides. These diseases are also known as primary ANCA-associated vasculitides. There are two major distinct subtypes of ANCA. One subtype is an antibody against myeloperoxidase (MPO), which stains in a perinuclear pattern (pANCA) by indirect immunofluorescence (IIF) using a neutrophil substrate, and the other subtype is an antibody against proteinase 3 (PR3), which stains in a diffuse granular cytoplasmic pattern (cANCA) by IIF. Enzyme-linked immunosorbent assay (ELISA) is used for target-specific ANCA determination [5]. MPO-ANCA can be found in MPA and AGA more frequently than in WG. On the other hand, PR3-ANCA is more specific in WG than the other primary systemic vasculitides. The association of MPA with MPO-ANCA is reported to be in the range of 40–80%, and MPA with MPO-ANCA is noted to be frequently associated with necrotizing glomerulonephritis and/or pulmonary capillaritis, namely, a pulmonary-renal syndrome similar to that observed in Goodpasture syndrome or WG [3, 4]. Furthermore, renal-limited vasculitis, which is characterized by pauci-immune focal necrotizing crescentic glomerulonephritis (pFNCGN), is classified as MPA using European Medicines Agency (EMEA) algorithm [6]. Interestingly, MPO-ANCA-associated vasculitides are more common in Asian countries than in the USA or Europe, where 80% of AAV is PR-3 ANCA [6, 7] and ANCA-positive, while anti-MPO-negative patients with MPA most often have antibody specificity for PR3 [3]. 

In Japan, MPO-ANCA has been reported to account for 90% of cases of AAV [8, 9]. Therefore, renal-limited AAV in Japan is almost exclusively MPA, whereas in the UK only 41% of cases of renal limited AAV have MPA, although the overall incidences of renal vasculitis are similar in the UK and Japan [6].

 On the other hand, ANCA has also been described in patients with rheumatic autoimmune diseases, including rheumatoid arthritis, systemic lupus erythematosus, Sjögren's syndrome, polymyositis/dermatomyositis, and antiphospholipid syndrome. The fluorescence patterns encountered in these diseases are mostly pANCA. A multitude of target antigens, including not only MPO but also lactoferrin, elastase, lysozyme, cathepsin G, and bactericidal/permeability-increasing protein (BPI) have been described in rheumatic autoimmune diseases [3, 10]. However, ANCA in SSc is uncommon, showing a positive rate ranging from 0 to 18% according to investigators [3, 11–17], in spite of the development of many autoantibodies, such as antitopoisomerase-I (Scl-70) antibodies, anticentromere antibodies, and rheumatoid factors, in SSc. ANCA in SSc usually shows pANCA and/or anti-MPO-ANCA, although BPI and cathepsin G were reported to be the major antigenic targets of ANCA seen in patients with SSc [18].

## 3. Characteristics of MPA

 MPA is clinically characterized by a multisystemic disease such as RPGN, alveolar hemorrhage, mononeuritis, and skin involvement, as well as other manifestations. It is also characterized by a high frequency of MPO-ANCA, showing predominant pANCA by IIF as mentioned above. Pathologically, inflammation and necrosis in various sites of vessels including arteries, arterioles, capillaries, and venules are observed, and pFNCGN characteristically occurs in the context of MPA as well as WG. The severity of the injury requires immediate treatment with immunosuppressive drugs including cyclophosphamide and corticosteroids.

 There are also some differences in the clinical manifestations of MPA, in addition to the clinical phenotype of renal AVV, between Europe and Japan.  Table 1 shows a comparison of clinical findings of MPA in the French Vasculitis Study Group reported by Guillevin et al. [4] and the Japanese Nationwide Epidemiological Survey [19] reported by Hashimoto et al. The observed differences might be expected because the same diagnostic criteria were not used, although the inclusion criteria for the diagnosis of MPA were almost the same.  Table 2 shows the diagnostic criteria of MPA used in the Japanese Nationwide Epidemiological Survey. Interestingly, the female : male sex ratio was 1.23 in the French study, which contrasts with 0.43 in the Japanese study, although the mean age at diagnosis was almost the same. The incidence of clinical manifestations including fever and weight loss in the French study was greater than that in the Japanese study, but the incidences of arthralgias and myalgias were almost the same. Concerning visceral involvement, the incidence of renal involvement and lung involvement, such as alveolar hemorrhage, pneumonitis, and pleuritis, in the Japanese study was greater than that in the French study; in contrast, the incidence of mononeuritis multiplex, central nervous system involvement, and gastrointestinal tract involvement in the French study was greater than that in the Japanese study. The frequencies of hypertension were 34.1% in the French study and 41.3% in the Japanese study.

 In the French study, ANCA was present in 74.5% of patients, of whom 86.8% had pANCA and the remainder had cANCA. In the Japanese study, pANCA was present in 90.4% of patients and cANCA was present in 9.6%. Among pathological findings in the Japanese study, necrotizing vasculitis was present in 32 out of 55 patients and crescentic glomerulonephritis was present in 41 out of 47 patients (not indicated in the French study). In the Japanese study, most of the patients were treated with corticosteroids (93.4%) and immunosuppressant drugs (71.0%). The mortality rate was 12.9% and the most frequent cause of death was infection (36.5%) followed by alveolar hemorrhage (17.3) and renal failure (13.5%). Guillevin et al. [4] noted that in the French study, deaths were less frequent when patients had been treated with corticosteroids and immunosuppressive drugs, but relapse of MPA was common.

## 4. AAV in SSc

 SSc is divided into two major clinical subsets, namely diffuse cutaneous and limited cutaneous disease, which are distinguished from one another primarily by the degree and extent of skin involvement [20]. Overlap syndrome, which has either diffuse or limited cutaneous disease and typical features of one or more of other connective tissue diseases, is also present [1]. Since the identification of antitopoisomerase-I (anti-Scl-70 antibody) and anticentromere antibodies, it has been well known that antitopoisomerase-1 antibodies are associated with diffuse cutaneous disease as well as evident renal involvement and pulmonary interstitial fibrosis, and anticentromere antibodies are associated with limited cutaneous disease as well as pulmonary hypertension. Anti-U1RNP and anti-Ku antibodies are observed in patients with overlap syndrome of SSc and myositis. Although the number of cases with overlap syndrome of SSc and MPA among the cases of SSc with AAV that have been reported is unknown, several cases of overlap syndrome were suggested to be present by Rho et al. [21] who analyzed and reviewed the clinical characteristics of SSc with AAV in 50 cases reported in 31 articles. In this study, MPO-ANCA was the predominant type (72%), but PR-3 ANCA was also found in some cases (24%). The authors noted that 33 out of 50 cases had definite features of AAV with pathological or clinical evidence including crescentic glomerulonephritis, RPGN, pulmonary hemorrhage, skin and/or nerve vasculitis, and necrotizing vasculitis of muscle. High levels of inflammatory activity, such as high ESR or CRP, or abnormal urinary sediments, were also indicated. Cases with MPO-ANCA had a higher prevalence of renal impairment and pulmonary hemorrhage than those with PR-3 ANCA. Having antitopoisomerase-1 antibodies made the development of AAV in SSc three times more likely than that in patients who had neither antibody. The mortality rate was 39.4% with virtually all of the deaths occurring within 1 year. The major causes of deaths included infection or septic shock, pulmonary hemorrhage, intracerebral hemorrhage due to coagulopathy, and acute cardiac failure. These findings strongly suggest the coexistence of MPA and strongly contrast with the survival rates and causes of deaths that are generally recorded for patients with SSc. 

 Although the association of normotensive rapid renal failure and MPO-ANCA that suggested the existence of a renal specific subset apart from scleroderma renal crisis was noted [13], nearly one-third of the MPA patients with renal involvement were hypertensive [4, 19], and hypertension was not an essential concomitant of renal failure due to SSc even though renal arterial stenosis was observed pathologically in SSc [22]. Blood pressure measurement cannot indicate whether or not scleroderma renal crisis or RPGN associated with AAV exists. However, the association of normotensive renal failure with microangiopathic hemolytic anemia in SSc was indicated [23]. When rapid renal failure or RPGN with active urine sediments or systemic inflammatory manifestations is observed in patients with SSc, the possibility of MPA should always be considered. In these cases, a renal biopsy should be considered to evaluate histological findings because the use of basic laboratory indicators, such as hematuria, proteinuria, or serum creatinine level, is considerably limited in facilitating the prediction of the site affected by vasculitis [2, 4, 24]. The characteristic renal histopathological findings of MPA are pauci-immune necrotizing and/or crescentic focal necrotizing glomerulonephritis as well as small vessel arteritis, which is identical to the lesions seen in WG or renal-limited vasculitis, which is a subtype of MPA [6, 9]. However, this finding distinguishes the disease from polyarteritis nodosa and SSc with fulminating course of malignant hypertension in which mucoid thickening of proximal interlobular arteries and fibrinoid necrosis in distal interlobular arterioles are specific for SSc [25].

 Mortality is greatest in the setting of pulmonary-renal syndrome in MPA. Pulmonary lesion is a small-vessel vasculitis, with fibrinoid necrosis of capillaries, leading to alveolar septal disruption, blood-filled alveoli, and the clinical sequelae of dyspnea, cough, and/or hemoptysis. The relative risk of death in MPA has been calculated to be 8 times higher in patients with pulmonary hemorrhage [26]. On the other hand, pulmonary hypertension, which is mostly observed in limited cutaneous scleroderma, is rare in MPA, although a few cases with WG and MPA accompanied with pulmonary hypertension have been reported [27]. In SSc, interstitial fibrosis was found to be the most common pulmonary lesion, showing bilateral fibrosis of lower lung or honeycomb lung in chest X-ray. Its presence correlated well with clinical measurements of restrictive lung disease and decreased diffusing capacity. Arteriolar thickening, described as medial hypertrophy or concentric intimal proliferation, was the most specific lesion in lungs, being noted in 29% of SSc patients in autopsy cases [22]. The differences in vascular clinical findings between SSc and MPA are shown in Table 3.

No treatment for SSc is proven to be effective in preventing progression of disease, reversing fibrosis, or improving long-term outcome, although a number of novel agents including anti-interleukin-6, transforming growth factor-*β*-directed therapies, and other novel biological agents are being developed [28]. However, if SSc patients with MPA have life-threatening visceral involvement such as rapid renal failure or RPGN and pulmonary hemorrhage, the patients should be treated with induction therapy using cyclophosphamide, methotrexate, corticosteroids, or plasmapheresis, and so forth, according to disease severity soon after the diagnosis of MPA, although attention should be paid to reducing treatment toxicity [29].

## 5. Significance of MPO-ANCA in Overlap Syndrome of MPA and SSc

 The etiology of MPA is unknown, but is generally considered to be the result of an interaction between triggering agents and disease susceptibility genes. In primary AAV, MPO-ANCA as well as PR3-ANCA has been established as a marker for diagnosis and has been implicated in the pathogenesis of vasculitis [2, 30]. In most patients with MPA and/or SSc with AAV, high titers of MPO-ANCA are associated with disease activity, rises in MPO-ANCA titers precede relapses, and even a case of MPO-ANCA seroconversion associated with fulminant vasculitis in antitopoisomerase-1 antibody positive SSc has been reported [31], but MPO-ANCA titers do not necessarily correlate with disease activity or vasculitis syndrome. There are also the cases without clinical manifestations related to AAV in spite of MPO-ANCA positivity. The same can be seen for other autoantibodies, such as anti-U1-RNP antibodies, antiphospholipid antibodies, and rheumatoid factor, in rheumatic diseases. Although the reason for cases without clinical manifestations showing MPO-ANCA positivity is not known, the following reasons may be considered: (1) it is a predictive marker for the development of AAV in the future, (2) it is an epiphenomenon, and (3) false positivity. The pathogenic potential of MPO-ANCA to small-vessel vasculitis may be due to not only quantities but also qualities of MPO-ANCA such as immunoglobulin phenotypes, affinity and/or avidity of MPO-ANCA, and specific risk epitope for MPO-ANCA. 

Concerning the source of antigens, attention has been focused on neutrophil extracellular traps (NETs)[32], which are chromatin fibers and are released by ANCA-stimulated neutrophils and contain the targeted autoantigens of PR3 and MPO [33] as well as topoisomerase-1. This may be circumstantial evidence that antitopoisomerase-1 antibodies occur more frequently in SSc with AAV than are usually found in SSc as described in the paper of Rho et al. [21]. 

 Some investigators pointed out that ANCA positivity in SSc is a red flag and draws attention [17, 21]. This is true, and it is important not to overlook characteristic clinical manifestations of AAV during the course of the disease in SSc.

## Figures and Tables

**Table 1:** Comparison of clinical manifestations in MPA between French and Japanese studies.

	French study [4]	Japanese study [19]
Cases	85	63
Age, mean (range) years	56.8 (16–86)	59.0 (13–91)
Sex ratio, Male : Female	47 versus 38	19 versus 44
Fever	55.30%	71.00%
Weight loss	72.90%	42.90%
Skin involvement	62.40%	24.20%
Arthralgias	50.60%	62.90%
Myalgias	48.20%	50.80%
Renal involvement	78.80%	87.30%
Renal insufficiency	(47/67) 70.1%	49.20%
Rapidly progressive GN	—	66.70%
Proteinuria	(54/67) 80.6%	93.10%
Hematuria	(45/67) 67.2%	76.70%
Lung involvement	24.70%	63.50%
Alveolar hemorrhage	11.80%	22.20%
Pneumonitis	10.60%	33.30%
Pleiritis	5.90%	19.00%
Mononeuritis multiplex	57.60%	30.00%
Central nervous system involvement	11.80%	6.30%
Gastrointestinal tract involvement	30.60%	6.30%
Hypertension	34.10%	41.30%
Myocardial infarction	2.40%	1.60%
Pericariditis	10.60%	3.20%
Cardiac failure	17.60%	3.20%

**Table 2:** Proposed diagnostic criteria for microscopic polyangiitis (MPA). (Research Committee on Intractable Vasculitis and Research Committee on Epidemiology of Intractable Diseases, The Ministry of Health and Welfare of Japan, 1998).

(1) Clinical manifestations
(i) rapidly progressive glomerulonephritis
(ii) pulmonary hemorrhage or interstitial pneumonia
(iii) organ involvement besides kidney and lung:
purpura, subcutaneous bleeding, gastrointestinal bleeding, mononeuritis multiplex, and so forth

(2) Histological findings
necrotizing vasculitis in capillaries, venules, or arterioles, with perivascular inflammatory infiltrate

(3) Laboratory findings
(i) positive MPO-ANCA
(ii) elevated level of CRP
(iii) proteinuria and/or hematuria, or elevated levels of BUN and/or creatinine
(iv) chest X-ray findings: infiltration (pulmonary hemorrhage), and/or interstitial pneumonitis

(4) Diagnosis
(i) Definite
(a) At least two clinical manifestations with the histological findings
(b) At least two clinical manifestations including items (i) or (ii), and positive MPO-ANCA
(ii) Probable
(a) At least three clinical manifestations
(b) One clinical manifestation and positive MPO-ANCA

(5) Exclusion diseases
(i) polyarteritis nodosa
(ii) Wegener's granulomatosis
(iii) allergic granulomatous angiits (Churg-Strauss syndrome)
(iv) Goodpasture syndrome

**Table 3:** Differences in vascular clinical findings between SSc and MPA.

	SSc	MPA
Raynaud's phenomenon	+	−
digital ischemia	+	−
skin ulcers	+	+
skin nodules	−	+
Purpura	−	+
nail bed change/telangiectasia	+	−
Myositis	+	+
interstitial pneumonia	+	+
honeycomb lung	+	−
alveolar hemorrhage	−	+
pulmonary hypertension	+	−
RPGN	−	+
scleroderma renal crisis	+	−
mononeuritis multiplex	−	+
CNS vasculitis	−	+
Histological findings;		
fibrinoid necrosis	arteries, arterioles	capillaries, arterioles
intimal hyperplasia	+	−
capillaritis	−	+
pFNCGN	−	+

SSc: scleroderma

MPA: microscopic polyangiitis

RPGN: rapidly progressive glomerulonephritis

CNS: central nervous system

pFNCGN: pauci-immune focal necrotizing crescentic glomerulonephritis.

## Abbreviations

AAV: ANCA associated vasculitis
AGA: Allergic granulomatosis angiitis
ANCA: Anti-neutrophil cytoplasmic antibody
BPI: Bactericidal/permeability-increasing protein
cANCA: Cytoplasmic ANCA
ELISA: Enzyme-linked immunosorbent assay
EMEA: European Medicines Agency
IIF: Indirect immunofluorescence
MPA: Microscopic polyangiitis
MPO: Myeloperoxidase
NETs: Neutrophil extracellular traps
pANCA: Perinuclear ANCA
pFNCGN: Pauci-immune focal necrotizing crescentic glomerulonephritis
RPGN: Rapidly progressive glomerulonephritis
SSc: Systemic sclerosis
WG: Wegener's granulomatosis.
